# Supplementary material for: Priorities and barriers for ageing well; results from stakeholder workshops in rural and urban Rwanda
Source: PLoS One. 2024 Apr 1;19(4):e0297299. doi: 10.1371/journal.pone.0297299 (PMC10984394; doi:10.1371/journal.pone.0297299)
Supplement: S1 Table — A full list of stakeholder organizations that were invited to participate in the workshops. (DOCX) [file pone.0297299.s001.docx]

**Supporting Information**

**S1 Table, Stakeholder organizations invited.**

| Kacyiru Stakeholders Organizations | Attended the workshop |
| --- | --- |
| Anglican Church of Rwanda/Kacyiru Sector | Yes |
| RSSB/Ejo Heza /Kacyiru Sector | Yes |
| Social Affairs/Kacyiru Sector | Yes |
| Health/Gasabo Sector (MOH representative) | Yes |
| St Dominican Church | Yes |
| Kamatamu Cell | Yes |
| Tearfund/Gasabo District | Yes |
| Kibaza Cell | Yes |
| Kamutwa Cell | Yes |
| Representative of the disabled/Kacyiru Sector | Yes |
| Women's representative | No |
| Joint Action Development Forum (JADF) - Kacyiru Secretary (Kacyiru) | No |
| Respresentative from the Sector/Kacyiru | No |
| Butaro Stakeholders Organizations | Attended the workshop |
| Muhotora Cell | Yes |
| Disabled Representative/Butaro Sector | Yes |
| Non-Communicable Diseases Representative/Butaro Sector | Yes |
| Private Sector Federation (PSF) Representative/Butaro Sector | Yes |
| Adventist Church/Butaro Sector | Yes |
| Anglican Church of Rwanda/Butaro | Yes |
| Social Affairs Burera District | Yes |
| Rusumo Cell | Yes |
| Women Representative/Butaro Sector | Yes |
| Representative Executive Secretary Butaro Sector | Yes |
| Gatsibo Cell | Yes |
| Rwanda Social Security Board (RSSB)/ Ejo Heza Representative/Butaro Sector | Yes |
| JADF Permanent Secretary/Burera District | Yes |
| BDH Representative - A doctor in palliative care/hospice care | No |
| Inshuti Mu Buzima (IMB) – Program on Social and Economic Rights (POSER) Program Manager | No |
