# Supplementary figures and images for: Priorities and barriers for ageing well; results from stakeholder workshops in rural and urban Rwanda
Source: PLoS One. 2024 Apr 1;19(4):e0297299. doi: 10.1371/journal.pone.0297299 (PMC10984394; doi:10.1371/journal.pone.0297299)

**S1 Figure. Complete responses from workshops.**


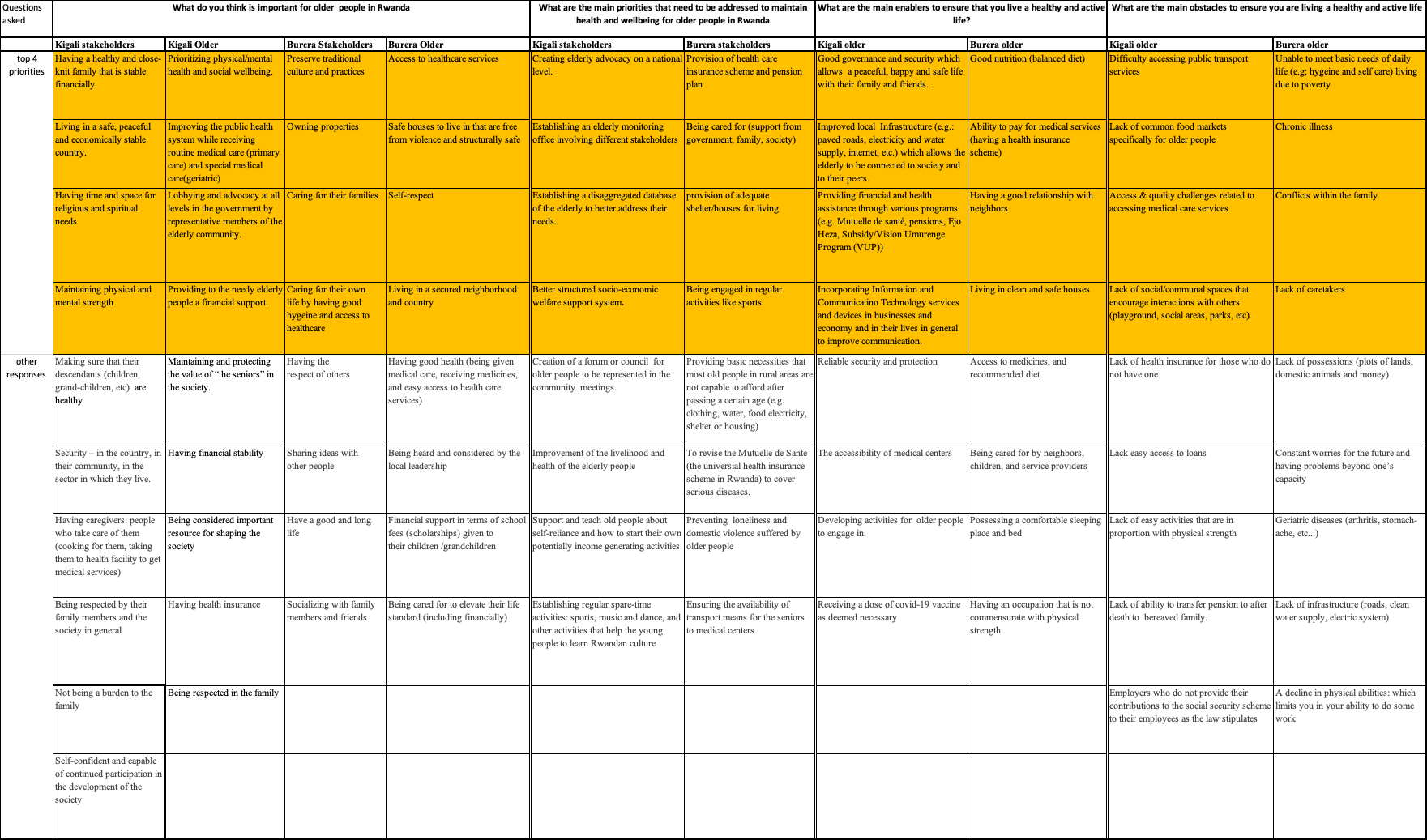

Supplement: S1 Fig — (DOCX) [file pone.0297299.s002.docx]
