# Supplementary material for: Priorities and barriers for ageing well; results from stakeholder workshops in rural and urban Rwanda
Source: PLoS One. 2024 Apr 1;19(4):e0297299. doi: 10.1371/journal.pone.0297299 (PMC10984394; doi:10.1371/journal.pone.0297299)
Supplement: Attachment — Submitted filename: Review Manuscript Number PONE-D-23-19881.docx [file pone.0297299.s004.docx]

# Review of manuscript

# **‘Priorities and barriers for ageing well; results from stakeholder workshops in rural and urban Rwanda’**

Thank you for letting me review this promising manuscript, which I truly enjoyed reading. I want to start with commending the authors for their work with listening to and highlighting the voices of the end users of a policy. I also enjoyed reading about how this was done, which I think will be useful for other researchers in the field.

In order for this paper to be published, I have some comments that I suggest the authors first consider.

1. The objectives are to “identify and compare the needs and priorities of older people and other stakeholders involved in caring for them in rural and urban areas of Rwanda”. I appreciate the comparison as this identifies the unique perspective of older people and provides a great argument for involving them, and not just stakeholders. However, in the results (and very clearly in the Conclusions), more focus lies on the comparisons that the identification. To read about the actual ‘needs and priorities’, the reader is referred to figures and tables, which are useful to display all information in detail, but as I was expecting to learn more about needs and priorities, I find myself lacking an overview of these. I suggest rewriting the result to add more overview of the needs expressed in the workshops.
2. The stakeholder group (under ‘Study population’) needs to be described more in detail. The table in the appendix is useful, however as a reader not familiar with the context I can’t get a good picture of what kind of organisations these are. It needs to be more clearly described in the text.
3. The “socio-ecological model” used is not properly referenced or introduced when first mentioned. Please add this.
4. Line 129: “results were translated after prioritization” – what does this mean?
5. Line 175-176: “Authors did not have access to information…” This statement is difficult to understand. I assume you saw their faces and heard their voices in the workshop, which would be information to identify someone. Did you mean that you did not record and/or save any personal information?
6. A technicality around Figure 3: In several places, two sections of text are placed in the same space, making it difficult to read.
