# Supplementary material for: Priorities and barriers for ageing well; results from stakeholder workshops in rural and urban Rwanda
Source: PLoS One. 2024 Apr 1;19(4):e0297299. doi: 10.1371/journal.pone.0297299 (PMC10984394; doi:10.1371/journal.pone.0297299)
Supplement: Attachment — Submitted filename: Response 2 Reviewers_Health ageing paper_Revised[73].docx [file pone.0297299.s005.docx]

Dear Editors and reviewers,

Thank-you very much for taking the time to review our paper. Thank you also for the positive comments on it. We have taken all comments into consideration when revising the paper, and our responses are documented below.

We look forward to hearing from you in due course.

Kind regards

Editor’s questions to reviewers

3. Have the authors made all data underlying the findings in their manuscript fully available?

The [PLOS Data policy](http://www.plosone.org/static/policies.action#sharing) requires authors to make all data underlying the findings described in their manuscript fully available without restriction, with rare exception (please refer to the Data Availability Statement in the manuscript PDF file). The data should be provided as part of the manuscript or its supporting information or deposited to a public repository. For example, in addition to summary statistics, the data points behind means, medians and variance measures should be available. If there are restrictions on publicly sharing data—e.g. participant privacy or use of data from a third party—those must be specified.

Reviewer #1: No

Reviewer #2: Yes

*Response: Reviewer 2 is correct, all the data that were collected in the workshops form part of the paper, they are thus available to all readers without restriction.*

Reviewer Comments to the Author

Reviewer #1: I have tremendously enjoyed this report, and I think it is making a great contribution to our knowledge that intercultural and contextual differences in perception of public healthcare deserve much more careful consideration.

The manuscript is well written, and concise.

**Response:** Thank-you for your positive comments

I do have a few concerns though:

While the nature of this research is qualitative, authors have not reported their findings with rigorous qualitative methods. This report pertains to observations from a workshop, serving as a semi-structured interview/focus group--but authors state that this was not a focus group and hence not recorded.

**Response:** We apologise if the text in the article was misleading and led to the expectation that this was a formal qualitative study involving focus group discussions which were recorded, transcribed, and analysed in the study. It was not; the methodology was based on a nominal group technique, delivered in a workshop setting with plenary and roundtable discussions, to reach consensus on lists of priorities. The outputs were lists of agreed priorities. We have used the nominal group technique as it is recognized to be a valuable method for engaging discussions within a group and getting rapid agreement on the relative importance of issues and solutions. We have gone through the manuscript and removed instances where we had introduced terms like “*qualitative*” and “*focus groups*”, which may introduce confusion. We have also added references for the nominal group technique method, to facilitate understanding.

Specifically, I would have liked to learn more about the selective sampling process, guided by stakeholders. What pool of potential participants was available, what percentage were invited to participate; and how were those selected? A Table to indicate characteristics of each participant's occupation, disability, etc. would help.

**Response:** Each stakeholder was asked to nominate elderly individuals from the list of those engaged in their services or who were known in their communities. We requested that stakeholders nominate older participants to represent a broad range of characteristics (e.g., those living with disabilities and chronic diseases, farmers, and those who worked in public or private services before retirement). Elderly people nominated by stakeholders were then listed in random order by district and cell (a subdivision of a sector, which is a subdivision of a district), and were called by phone until the required number confirmed their availability. From each cell at least 2-3 people were selected such that a total of around 15 elderly individuals were invited in each study area (Kigali and Burera), These participants were retirees, but they represented a diverse set of characteristics, as detailed in lines 121 – 123 of the manuscript.

We have included the above text in the manuscript to make this clearer.

Also, the dynamics through which individuals reached a consensus on the single priority needs to be explained. It is natural to expect disagreements existing (and if not, this should be reported too.) and the reader would learn from attention to such. (it's understood that the authors were blind to which participant said what.) A few quotations or specific examples can help.

**Response:** The nominal group technique focuses on the nomination of ideas by participants at roundtables. Discussion was encouraged in the roundtable and plenary sessions prior to prioritizing the lists, such that the prioritization was informed by the prior discussion. Hence, disagreements on the selected priorities or their orders are uncommon. Where disagreements occurred, facilitators worked with the discussion group to improve agreement. The final list was selected by voting by all participants, and disagreement on the order after voting is unusual in nominal group technique methods.

We have added this text to the manuscript to improve clarity around resolution of disagreements.

It is not clear how stakeholder and older participants' responses were summarized (since they were not recorded). Who did the summarization? What were the criteria? The same applies to the process of categorization.

**Response:** Please also see responses above. The methodology used was nominal group technique, which involves discussion to orient individuals to the issues under consideration followed by the capture of lists of priorities. The facilitators captured the lists from the participants on round tables, then after discussion, the participants were asked to rank their list. The ranking was done by facilitated discussion, then there was voting in plenary on the lists put forwards by round tables. There was some summarization which occurred in the combination of responses from the multiple roundtables such that the lists presented for voting were free of duplicates. This was done by agreement of the facilitators of the meeting.

We have added clarity to the manuscript that prior to voting in plenary, the facilitators of the meeting discussed the lists provided by roundtables and remove duplicate ideas.

**The text now reads:** *“Following the discussion, the facilitators combined the top 4 responses from each of the small groups after discussing amongst the facilitator group which were duplicate responses and removing these. All stakeholders then voted for the top 4 priorities for each question; facilitators recorded these priorities.”*

Were the four categories pre-determined? Were they implied in the questions? Were they deduced from the summaries? If so, were the responses open-coded? Please refer to a standard qualitative methodology to describe your approach, so others can replicate the effort in other contexts.

**Response:** Yes, the four categories were predetermined and implied in the questions. As per above responses, this was not a qualitative study, and no coding was done.

Please rephrase statements such as "we tended towards a deterministic approach" to not confuse readers with philosophical connotations. I think in explaining this "deterministic approach" it is important to orient the user about existing healthcare infrastructure for older care in Rwanda--what "externalities" exist?

**Response**: We apologise, the manuscript should have read that we used the philosophy of determinism in our analysis, when categorizing responses in the socio ecological model. We have adjusted the text changing *“we tended towards a deterministic approach”* and to *“we were guided by philosophy of determinism”* It is challenging in practice to know the balance between whether behavior is medicated by individual’s agency or externalities. The philosophy of determinism suggests that most events or behavior are governed by externalities. This philosophy has gained traction in individual health, especially in high income countries and in particular around making healthy choices. Drivers of choices in Rwanda, whether predominately through agency or externalities has been understudied. However, on discussion, the authors agreed that in many cases, the existence of externalities was more likely to influence decisions and choices than agency, for the issues under discussion.

We have included the above text in the limitations of the manuscript.

Additionally in the discussion, we have added some context on healthcare access in Rwanda, as follows: *Older people in Rwanda have the right to health and are supported in acquiring medical insurance like Mutuelle de Sante, receiving social support (VUP), and accessing the pension system through the Rwanda Social Support Board (RSSB). These elements were considered existing externalities in our classification using the socio-ecological model.*

Reviewer #2: Thank you for letting me review this promising manuscript, which I truly enjoyed reading. I want to start with commending the authors for their work with listening to and highlighting the voices of the end users of a policy. I also enjoyed reading about how this was done, which I think will be useful for other researchers in the field.

**Response:** Thank-you very much for these positive comments

In order for this paper to be published, I have some comments that I suggest the authors first consider.

1. The objectives are to “identify and compare the needs and priorities of older people and other stakeholders involved in caring for them in rural and urban areas of Rwanda”. I appreciate the comparison as this identifies the unique perspective of older people and provides a great argument for involving them, and not just stakeholders. However, in the results (and very clearly in the Conclusions), more focus lies on the comparisons that the identification. To read about the actual ‘needs and priorities’, the reader is referred to figures and tables, which are useful to display all information in detail, but as I was expecting to learn more about needs and priorities, I find myself lacking an overview of these. I suggest rewriting the result to add more overview of the needs expressed in the workshops.

**Response:** We have added some general description of the results from each group, in addition to the comparisons.

We have added the following text: *“The results from both workshops, highlighted that it is important to older people to prioritise physical and mental health, improve the health system and provide access to healthcare services, and provide safe housing. Stakeholders in both workshops felt it was important for older people to have a healthy and close-knit family, be financially stable, live in a peaceful country, preserve culture and tradition and own properties. The priorities that need to be addressed, put forwards by stakeholders, include creating advocacy for the elderly at a national level, establishing an elderly monitoring office, the provision of a healthcare insurance scheme and a pension plan, and being cared for by government and family. The main enablers that older people put forwards to help them maintain health and well-being were good governance and security, improved local infrastructure, good nutrition, and ability to pay for medical services. The main obstacles that older people put forwards were difficulty accessing transport services, lack of food markets specifically for older people, inability to meet their basic needs due to poverty, and chronic illness.”*

2. The stakeholder group (under ‘Study population’) needs to be described more in detail. The table in the appendix is useful, however as a reader not familiar with the context I can’t get a good picture of what kind of organizations these are. It needs to be more clearly described in the text.

**Response:** The organizations invited as stakeholders included charitable, civil society, government, and religious groups that work closely with the elderly in Rwanda by providing services or care. For each type, at least 1-2 representatives were invited. Out of the 28 invited, 23 attended from both Burera and Kigali.

We have added this text in the manuscript to improve the clarity.

3. The “socio-ecological model” used is not properly referenced or introduced when first mentioned. Please add this.

**Response:** We have adjusted the referencing for the SEM and added some text for clarity when we first mention is as follows: *“The social-ecological model (SEM) is a framework advanced by the World Health Organization. It aids in understanding the diverse influences on an individual's health and well-being, including Individual, Relationships, Environmental, and Social or Community levels.”* The reference is provided; see reference number 11.

4. Line 129: “results were translated after prioritization” – what does this mean?

**Response:** We conducted all the workshops and ranked the priorities from the discussions in Kinyarwanda (local language). The Rwanda team translated the priorities into English, and Rwanda and English-speaking authors discussed the translation to make sure that the meaning was clear in English and accurate in Kinyarwanda.

5. Line 175-176: “Authors did not have access to information…” This statement is difficult to understand. I assume you saw their faces and heard their voices in the workshop, which would be information to identify someone. Did you mean that you did not record and/or save any personal information?

**Response:** We reviewed the entire document and did not find the statement, “Authors did not have access to information…”. In the workshop, only four authors facilitated the discussions; however, no recordings were made, and no personal data were saved.
