# Supplementary material for: Priorities and barriers for ageing well; results from stakeholder workshops in rural and urban Rwanda
Source: PLoS One. 2024 Apr 1;19(4):e0297299. doi: 10.1371/journal.pone.0297299 (PMC10984394; doi:10.1371/journal.pone.0297299)
Supplement: Attachment — Submitted filename: Reviewer 2 comments round 2.docx [file pone.0297299.s006.docx]

Thank you for inviting me to re-review this manuscript. I want to acknowledge that the authors have indeed made improvement to the manuscript, and that this has made it clearer and more aligned with the stated aim.

I suggest a few minor adjustments to get this manuscript in order for publication.

- The aims in the abstract and in the end of the introduction are different, not just in phrasing but in a key concept: whether you aimed to compare or not. This needs to be aligned.
- There is a need to oversee the writing in the revised parts of the manuscript (perhaps using ‘track changes’ has been difficult?). For example, ‘Discussion’ is written two times and some sentences are written in a way that they repeat themselves. Another example is lines 132-134 which need to be fixed, the sentence is not understandable.
- I get what the ‘top responses’ mean as there was a prioritization process, but what is the ‘first four responses? There is a mix-up between the concepts in the manuscript (for example line 245 refer ‘the first four’ in table 1, but this table shows ‘top fours priorities’) which makes me assume that it is actually the same thing. These terms need to be overseen.
- This has not been amended since I first reviewed this manuscript: In Figure 3, in several places, two sections of text are placed in the same space, making it difficult to read.

Thank you again for a great manuscript!
