# Supplementary material for: Priorities and barriers for ageing well; results from stakeholder workshops in rural and urban Rwanda
Source: PLoS One. 2024 Apr 1;19(4):e0297299. doi: 10.1371/journal.pone.0297299 (PMC10984394; doi:10.1371/journal.pone.0297299)
Supplement: Attachment — Submitted filename: R2R_Health ageing paper_Dec2023.docx [file pone.0297299.s007.docx]

Dear Editors and reviewers,

Thank-you very much for taking the time to review our paper. Thank you also for the positive comments on it. We have taken all comments into consideration when revising the paper, and our responses are documented below.

We look forward to hearing from you in due course.

Kind regards

Theogene on behalf of the authors

Reviewer Comments to the Author

Reviewer's Responses to Questions

**Comments to the Author**

1. If the authors have adequately addressed your comments raised in a previous round of review and you feel that this manuscript is now acceptable for publication, you may indicate that here to bypass the “Comments to the Author” section, enter your conflict of interest statement in the “Confidential to Editor” section, and submit your "Accept" recommendation.

Reviewer #1: All comments have been addressed.

Reviewer #2: (No Response)

2. Is the manuscript technically sound, and do the data support the conclusions?

Reviewer #1: Yes

Reviewer #2: Yes

3. Has the statistical analysis been performed appropriately and rigorously?

Reviewer #1: N/A

Reviewer #2: N/A

4. Have the authors made all data underlying the findings in their manuscript fully available?

Reviewer #1: Yes

Reviewer #2: Yes

5. Is the manuscript presented in an intelligible fashion and written in standard English?

Reviewer #1: No

Reviewer #2: No

6. Review Comments to the Author

Please use the space provided to explain your answers to the questions above. You may also include additional comments for the author, including concerns about dual publication, research ethics, or publication ethics. (Please upload your review as an attachment if it exceeds 20,000 characters)

Reviewer #1: Thank you for addressing the comments.

I think a bit more work will make your paper more poignant.

1. Thank you for explain your SEM and the determinism philosophy, but they need to be a bit more cohesively explained and tied into your research method which is nominal group technique. Use philosophy to explain your theoretical mode that inform the research you want to do and necessitates the research method you have chosen.

If determinism is the philosophical ground for this study, then it needs to be stated as such in the introduction, and then it need to be explained how the socioecological model fits within this philosophical framework, and THEN explain why the nominal group technique was suitable to test the socio-ecological model that was informed by your determinism philosophy.

**Response to reviewer:**

Thank you, for the constructive comments. we have revised our manuscript to more explicitly state that our study is grounded in the philosophy of determinism. This is now clearly articulated in the introduction, highlighting how determinism views the aging process as significantly influenced by external societal and environmental factors and aligning with the socio-ecological model. The following statement was added in the manuscript: *“To achieve this, we rely on the philosophy of determinism, which suggests that the conditions and experiences of persons – including older adults in Rwanda - are primarily shaped by external societal and environmental factors, rather than individual agency alone. To further categorize and analyze our findings, we use the socio-ecological model (SEM) as a framework. This model allows for a comprehensive understanding of the multiple influences on an individual's health and well-being, including individual, relational, environmental, and societal factors.” Line 99 – 109.*

In the methods section, we have also stated how nominal group technique is suitable to test SEM and determinism, by adding the following statement: “*This method is consistent with our determinist approach, as it allows for the articulation of the influence of external societal and environmental factors on the individual, relational, environmental, and societal perspectives of both older people and stakeholders. Line 166 – 168.*

2. There are also typos such as behavior is mediCated--created by autocorrect.

**Response to reviewer:**

We have gone through the manuscript and corrected any errors that we found, we have also made several small edits to improve the readability of the manuscript.

Reviewer #2: Thank you for inviting me to re-review this manuscript. I want to acknowledge that the authors have indeed made improvement to the manuscript, and that this has made it clearer and more aligned with the stated aim.

I suggest a few minor adjustments to get this manuscript in order for publication.

• The aims in the abstract and in the end of the introduction are different, not just in phrasing but in a key concept: whether you aimed to compare or not. This needs to be aligned.

**Response to reviewer:** This has been addressed. The aim in both places now reads: “*This study aimed to identify and compare the needs and priorities of older people and other stakeholders involved in caring for them in rural and urban areas of Rwanda.”*

• There is a need to oversee the writing in the revised parts of the manuscript (perhaps using ‘track changes’ has been difficult?). For example, ‘Discussion’ is written two times and some sentences are written in a way that they repeat themselves. Another example is lines 132-134 which need to be fixed, the sentence is not understandable.

**Response to reviewer:** This was addressed. Lines 156 -160 now read: “*All communication was conducted in Kinyarwanda, the local language, and results were translated into English for analysis. Translations into English were done by the Rwanda team, and both Rwandan and English-speaking authors then discussed the translation to ensure that the meaning was clear in English and remained accurate in Kinyarwanda.”*

• I get what the ‘top responses’ mean as there was a prioritization process, but what is the ‘first four responses? There is a mix-up between the concepts in the manuscript (for example line 245 refer ‘the first four’ in table 1, but this table shows ‘top fours priorities’) which makes me assume that it is actually the same thing. These terms need to be overseen.

**Response to reviewer:** This has been addressed. The manuscript was revised to consistently use the terms “top four priorities” and “top four responses”, replacing “first four”. We hope that it is now clear.

• This has not been amended since I first reviewed this manuscript: In Figure 3, in several places, two sections of text are placed in the same space, making it difficult to read.

**Response to reviewer:** This has been addressed. Adjustments were made to the text size and positions to enhance readability.

Thank you again for a great manuscript!

**Response to reviewer: Thank-you for your positive comment!**
